# Extended factorial validity of the Capacity to Love Inventory

**DOI:** 10.3389/fpsyg.2026.1831380

**Published:** 2026-05-21

**Authors:** Aija de Starceva-Apele, Malgožata Raščevska, Nestor D. Kapusta

**Affiliations:** 1 Department of Psychology, Faculty of Educational Sciences and Psychology, University of Latvia, Riga, Latvia; 2 Department for Psychoanalysis and Psychotherapy, Medical University of Vienna, Vienna, Austria

**Keywords:** Capacity to Love Inventory, emotional intimacy, emotional regulation, factorial validity, loss, reliability, sexual desire

## Abstract

This study extends the factorial validity of the Capacity to Love Inventory (CTL-I) by using a version adapted to the Latvian context, inviting clinicians to use this construct to deepen their understanding of clinical problems. It aims to examine the reliability and several valid factor models of the CTL-I after its adaptation to a Latvian sample. The total number of participants was 649 (79% female, 20% male, 1% other), aged from 20 to 60 years. Results for the subscales and total scale reliability of the Latvian version of the CTL-I showed adequate to excellent levels, comparable to the original version (after removing three psychometrically problematic items). A six-factor confirmatory factor analysis (CFA) and a second-order two-factor exploratory factor analysis (EFA) and CFA model achieved acceptable goodness-of-fit indices in the Latvian sample. The new latent second-level factors could be called (1) emotional investment and intimacy and (2) regulation of sexual desire and loss. The results support the CTL-I as a scientifically grounded tool for assessing one’s capacity to engage in and sustain romantic relationships, with promising future applications for personality trait assessment, clinical assessment, treatment prognosis, and planning. Supporting one’s capacity to build emotional and intimate relationships and regulate sexual desires is an important social focus in which it is beneficial to invest more at a preventive level.

## 1 Introduction

This research joins the branch of quantitative research to operationalize the seemingly intangible matter of one’s capacity to love, measured along a continuum from the capacity to love (CTL) to limited capacity to love, developed within a psychoanalytical framework by means of the Capacity to Love Inventory (CTL-I; Kapusta et al., 2018). Prior to the quantification of the CTL-I, Otto Kernberg (1974, 1977, 2011) formulated a theoretical framework of the capacities involved in maintaining a sustainable romantic partnership through a contrast with their noticeable absence in various mental illnesses, especially conditions of personality pathology. The present study aimed to evaluate the internal consistency of the CTL-I construct and to test the psychometric validity of the original factor structure in a Latvian non-clinical sample, while also contributing to further development of its factor model.

CTL as a mental health indicator in clinical-theoretical writings was first mentioned by Sigmund Freud (1959), who cited recovered CTL as an indicator of the successful psychotherapeutic cure. Conversely, the loss of CTL was listed as one of the symptoms of depression, then called melancholia (Freud, 1957). Ironically, although the CTL theoretical framework has long been useful for clinicians practicing psychodynamic therapy, quantitative research on psychodynamic perspectives of romantic love–specifically through the development of the CTL-I (Kapusta et al., 2018)–did not emerge until more than a century later. Thus, the authors of the current study seek to make an additional contribution to addressing a quantitative research gap by providing empirical evidence on the constructs of romantic love, as well as supporting the quantitative investigation of psychodynamic and psychoanalytic approaches.

The original study of the CTL-I (Kapusta et al., 2018) outlines the process of psychometric modeling, tracing the development of the inventory from clinical–theoretical foundations to a psychometrically validated measure. Over the past decades, numerous psychometrically validated instruments have been developed to measure romantic love, facilitating its operationalization and advancing the understanding of the multifaceted nature of human romantic relationships. Prominent examples include the Sternberg Triangular Love Scale (Sternberg, 1986), the Love and Liking Scale (Rubin, 1970), the Passionate Love Scale (Hatfield and Sprecher, 1986), the Love Attitudes Scale (Hendrick, 1988), the Relationship Assessment Scale (Hendrick, 1988) and its revised version (Quispilaya-Capcha et al., 2024). However, the CTL-I offers a different perspective on romantic love by focusing on clinically sensitive personality dimensions. Further investigation of its underlying factor structure, especially through more comprehensive models, may yield new insights for both general and clinical applications.

The current study derives evidence-based conclusions about CTL in one more cultural environment to enable the CTL-I to be used, first, as a diagnostic tool for a person’s particular personality traits (capacity to love romantically) and, second, as an assessment tool for a couple’s romantic relationship quality. The development and further exploration of the CTL-I construct is also important as a precedent for the examination of psychological constructs that are seemingly not obviously related to clinical disorder categories but whose further exploration in samples suffering from specific types of disorders illuminates the emotional obstacles to the satisfaction of existential needs in one’s personal life and thus provides practical utility for clinical work.

The CTL-I is an instrument for assessing personality facets that not only explain success or failure in establishing and sustaining romantic relationships but can also be used as markers for assessing certain aspects of a person’s emotional development. Kernberg (1977) states that the capacity to fall and to remain in love reflects the successful completion of developmental tasks acquired cumulatively. Namely, (1) if the capacity for sensuous stimulation of erotogenic zones is integrated early, it serves as (2) the basis for the capacity for establishing stable and deep object relations and for acquiring the capacity for sexual love as a complex emotional disposition that integrates sexual excitement, tenderness, sexual identification, mature idealizations, and commitment (Kernberg, 1977). This work extends existing research on the CTL and provides additional, in-depth scientific evidence on the factor structure of the CTL-I, based on data from a Latvian sample. Therefore, we first scrutinized the content of the CTL scales, the formulations of their items, and the relevant psychometric properties reported in previous studies.

A person’s capacity to love romantically can be defined as a personality trait (Kapusta et al., 2018), constituted by a set of six latent facets: interest in the other (INT), basic trust (BTR), humility and gratitude (GRA), common ego ideal (CEI), permanence of sexual passion (PSP), and loss and mourning (LOM). The factor structure of the CTL-I has been shown to be cross-culturally robust in German and Polish (Kapusta et al., 2018), Italian (Margherita et al., 2018), Portuguese (Fonte et al., 2022), and Slovenian populations (Poštuvan et al., 2022). In all previous CTL studies, the construct developed by Kapusta et al. (2018) has been psychometrically demonstrated to consist of six factors or lower-order CTL dimensions at the first level, which together constitute the main factor (CTL). In the Austrian and Polish samples, all six factors demonstrated a psychometrically sound structure and contributed equally to the determination of CTL (Kapusta et al., 2018). The same factor structure was replicated in Italian and Slovenian studies that examined the construct’s factorial validity (Margherita et al., 2018; Poštuvan et al., 2022), and the six-factor model was also reported for the Portuguese version (Fonte et al., 2022, 2025). The Latvian adaptation of the CTL-I will facilitate cross-cultural research, which is essential for establishing a psychometrically robust construct across diverse sociocultural contexts. Latvia represents a unique setting that lies outside the core Central European cultural sphere, characterized by a complex sociocultural landscape shaped by its post-totalitarian history, ongoing adaptation to a capitalist system, and the coexistence of multiple religious traditions.

The level of factor loadings obtained in confirmatory factor analysis (CFA), previous studies reveal that the INT scale shows lower and more diverse factor loadings–items 1 and 7 appear to have repeatedly lower factor weights in the Polish, Austrian, and Italian samples. Lower factor weights are also reported for BTR scale items 13 and 15 in the Polish, Italian, and Slovenian study samples and item 15 in the Austrian sample. The LOM scale shows lower factor weights compared to the average across other CTL-I scales in the Austrian and Italian samples. The internal consistency of CTL-I scales in previous research ranged from 0.73–0.86 (Austria), 0.72–0.87 (Poland), 0.83–0.87 (Italy), 0.67–0.85 (Slovenia), and 0.72–0.88 and 0.67–0.90 (Portugal). Across all samples, Cronbach’s *α* ranged from 0.88 to 0.93. Thus, previous evidence suggests that the internal consistency of CTL-I scales reached acceptable, good, and excellent levels of agreement. The lowest reported reliability coefficients were for the INT and LOM scales, reported as acceptable by Kapusta et al. (2018), in contrast to good levels of fit in the other scales, while the INT scale achieved only an acceptable level of fit in the Slovenian sample andanother Austrian-German sample (Fuchshuber et al., 2025). These data suggest that the items that constitute CTL scales function differently across cultural environments, while certain items also seem to be repeatedly responsible for lowering the scale’s internal consistency.

To gain a deeper understanding of its latent structure, previous analysis on the CTL-I used a loop of inter-scale correlations. The data from correlation analyses obtained in previous studies reveal important realities. In the Austrian and Polish samples, INT, GRA, and CEI show no statistically significant relations with LOM, whereas LOM and BTR show diverse relations: a statistically significant positive relation in the Austrian sample (*p* < 0.001) and none in the Polish sample. Conversely, the PSP scale correlated significantly with all other CTL-I subscales (Kapusta et al., 2018). A similar pattern of correlations (*p* < 0.01) is reported for the Slovenian sample (Poštuvan et al., 2022). In the Italian sample, all CTL-I scales show positive statistically significant correlations; however, while those between INT, BTR, GTR, and CEI are at the level of *p* < 0.01, their relations with PSP and LOM are only at the level of *p* < 0.05. The tendency for the correlation coefficients of the INT, BTR, GRA, and CEI scales to build closer relations between them yet lower or no relations with PSP and LOM is borne out by previous research (Kapusta et al., 2018; Margherita et al., 2018; Poštuvan et al., 2022). In other countries, PSP and LOM show weak (statistically significant) mutual correlations, but they mostly remain higher than correlations with other scales. Thus, two groups of CTL-I scales emerged, with stronger ingroup correlations and weaker outgroup correlations. This allowed the authors to assume the possibility of another second-level factor structure of CTL-I beyond a unifactor model. However, to the best of current knowledge, prior CTL-I research has not examined whether a second-order factor structure exists. Similarly, there appears to be no empirical support for alternative CTL factor models, including both first-order and second-order structures.

Accordingly, the first task was to determine whether the original six-factor structure would be repeated in the Latvian sample and whether the CTL-I would achieve sufficient internal consistency. The second task was to scrutinize whether the latent relations among the six CTL-I scales would reveal any second-order two-factor solutions. This hypothesis was partly based on the analysis of the content of both groups of CTL-I scales. The first four CTL-I scales cover the themes of the capacity to experience emotional proximity, common values, and intimacy. PSP is specifically devoted to the maintenance of sexual passion, whereas the LOM scale covers emotionally challenging aspects of non-answered love situations, dealing with the aftermath of separation, and dealing with feelings of guilt and jealousy. Taken together, PSP and LOM characterize the regulation of sexual desire and loss.

Given that the developed theoretical model of the CTL-I has already been consistently validated in different cultural contexts (in Poland, Austria, Portugal, Italy and Slovenia), CFA was used as an appropriate approach to test the factor structure in the Latvian sample. Kline (2023) noted that CFA is particularly suitable for testing theoretically grounded and psychometrically validated measurement models. In contrast, the second-order factor structure of the CTL-I has not been previously studied in any cultural context. Therefore, both approaches were used to clarify the second-order factor structure, starting with exploratory factor analysis (EFA), followed by CFA to confirm the identified model. The development of the CTL-I factor models began with the testing and analysis of the test items and the psychometric indicators of the 6 scales in order to exclude those items (if any) that would not be suitable in the Latvian cultural environment both in terms of content and psychometrics.

From a psychometric perspective, examining higher-order factor models provides an important means of determining whether the covariance among first-order factors can be explained by more general underlying dimensions, thereby enhancing the parsimony and structural validity of the measurement model. From a clinical and personality psychology perspective, clarifying the higher-order organization of the construct—following the establishment of convergent and discriminant validity (which was not the aim of the current study)—may contribute to a more precise understanding of the functional domains of CTL, with potential implications for clinical application. Accordingly, investigating the second-order factor structure of the CTL-I represents an important step toward refining its theoretical foundation and clinical utility.

In light of this, the following research questions were formulated for the current study:

- Is the reliability of the Latvian-language version of the CTL-I similar to the original?
- Will the original six-factor structure of the CTL-I be confirmed in the Latvian population with acceptable values of goodness-of-fit indices?
- Will the six-factor CTL-I model also reveal a second-order two-factor solution in the Latvian sample with adequate psychometric criteria of model fit?

## 2 Materials and methods

### 2.1 Participants

The total number of participants included in the analysis was 649. Three respondents (0.46%) were excluded due to the high percentage of missing values. The age range was 20 to 60, and the number of female participants exceeded that of male and other genders (78.7%–female, 20.5%–male, 0.2%–other, 0.6%–did not indicate). The gender distribution of the Latvian sample is similar to that of the original study (Austrian: *n* = 547, 82.1% female; Polish: *n* = 240, 82.9% female). The age range of participants from the original sample was 16–66 in the Austrian sample (M = 28.92, SD = 10.22) and 18–50 in the Polish sample (*M* = 23.24, SD = 4.21). Thus, although there are differences between the Austrian, Polish, and Latvian samples, the mean age of the Latvian sample (*M* = 27.89, SD = 9.65) is close to that of the Austrian sample.

The population of Latvia is approximately 1.8 million. Sufficient Latvian language skills were a necessary precondition for participation in the research. The study sample comprised 441 (68%) students from four universities and 208 (32%) employees from other environments (state and local government institutions and businesses). This sample is characteristic of the general population. Data were collected in several stages between 2022 and 2024: 60% (390) of respondents completed paper questionnaires, whereas 40% (259) preferred the electronic questionnaire on Google Forms.

To achieve desirable psychometric outcomes for the factor models, the research sample was calculated according to the rule, where N (sample size) is estimated by the proportion of q (proportion between model variables and number of items): N/q ≥ 10, namely 649/41 items = 15.8 > 10. Although the current Latvian study does not fully replicate the distribution of the original research sample’s sociodemographic characteristics, it is nevertheless similar, and a wide spectrum of education levels was covered (see Table 1).

**Table 1:** Descriptive statistics of participants’ sociodemographic characteristics (*N* = 649).

Sociodemographic characteristics of participants	Number (percentage) or M (SD)
Age (range: 20–60 years)	27.89 (9.65)
Gender	
Female	511 (78.7%)
Male	133 (20.5%)
Did not indicate or other	4 (0.6%) and 1 (0.2%)
Education	
Primary	2 (0.3%)
Secondary	87 (13.4%)
Professional with secondary	91 (14.0%)
College	183 (28.2%)
Bachelor	210 (32.4%)
Master’s or higher	76 (11.7%)

### 2.2 Measures

Capacity to love was measured with the CTL-I (Kapusta et al., 2018), which was adapted for the Latvian-speaking population in this study. The CTL-I is a self-assessment questionnaire that measures six factors (described above). Every statement is evaluated on a 4-point Likert scale from 1 (totally disagree) to 4 (totally agree). Items 7, 13, 15, and 32–41 should be reversed. The original version of the CTL-I consists of 41 items; however, items 13 and 15 of the Latvian version revealed high cross-loadings with several CTL-I factors, while item 7’s total correlation coefficient with other items of the same scale was low. Therefore, these items were excluded (see Table 2 for the internal consistency of each CTL-I scale).

**Table 2:** Descriptive statistics of CTL-I scales in Latvian, Austrian, and Polish samples.

Scale	Number of items	M (SD)	Mdn	K-S p^a^	Cronbach’s α
First-order scales					
Interest in the other (INT)	7/6	20.36 (2.49)	21.00	<0.001	0.62^b^/0.73^c^/0.73^d^/0.72^e^
Basic trust (BTR)	9/7	22.56 (3.59)	23.00	<0.001	0.76^b^/0.81^c^/0.86^d^/0.83^e^
Gratitude (GRA)	7	20.61 (2.79)	21.00	<0.001	0.82^b^/.82^c^/0.81^d^/0.80^e^
Common ego ideal (CEI)	7	26.43 (3.67)	27.00	<0.001	0.81^b^/0.81^c^/0.81^d^/0.82^e^
Permanence of sexual passion (PSP)	2	5.28 (1.64)	6.00	<0.001	0.83^b^/0.83^c^/0.83^d^/87^e^
Loss and mourning (LOM)	8	20.48 (7.31)	21.00	<0.001	0.92^b^/0.92^c^/0.75^d^/0.72^e^
CTL-Total	41/38	115.71(13.73)	116.00	<0.001	0.90^b^/0.89^c^/0.90^d^/0.88^e^
Second-order scales					
Emotional investment and intimacy (EII)	28	89.96 (10.26)	92.00	<0.001	0.91^b^
Regulation of sexual desire and loss (RSDL)	10	25.76 (8.36)	26.00	<0.001	0.92^b^

^a^Kolmogorov–Smirnov test with Lilliefors criteria.

^b^Cronbach’s a values of CTL-I 38-item version.

^c^Cronbach’s a values of CTL-I 41-item version.

^d^Cronbach’s a coefficient in Austrian sample (Kapusta et al., 2018).

^e^Cronbach’s α coefficient in Polish sample (Kapusta et al., 2018).

### 2.3 Procedure

Our study was accepted by the Ethical Committee of the Department of Psychology, Faculty of Educational Sciences and Psychology, University of Latvia (Protocol No. 1/24.04.2020). Following *The Guidelines for Translating and Adapting Tests* (International Test Commission, 2017), both the original German-language version and the English-language version (used in the final publication) were translated into Latvian by independent translators who were not previously familiar with the CTL-I.

Two native Latvian speakers, who are professional translators specializing in English and German, were involved in translating both versions. The original Polish version was translated by one of the authors of the current research (M. Raščevska) in order to promote sensitivity to linguistic and cultural nuances, as suggested in *The Guidelines for Translating and Adapting Tests*. The authors of this research compared all three translations, discussed the nuances from the perspective of precise content and well-sounding wording, and agreed on the most equivocal formulations. A small pilot study with a group of 12 participants was undertaken, and after their feedback was analyzed, some corrections to the wording of certain statements were made. Finally, the translators provided back-translations from German and English, which were then approved by the authors of the current research.

Before gathering the data, the responsible authorities of each setting were informed of and agreed to certain ethical obligations: voluntary participation, confidentiality, anonymity, and the use of data only for the purposes of this research. The researchers’ ethical obligations (confidentiality, anonymity, use of data, etc.) were also explained to all participants.

### 2.4 Data analysis

SPSS (25.0) was used for data processing and statistical analysis, while JASP (0.95.4) was used for the confirmatory factor analysis (CFA), consistent with prior research, was carried out using JASP (version 0.95.4). Since the data did not fulfil normal distribution criteria (see Table 2), non-parametric methods (Spearman correlations) were used. To examine whether the underlying structure of our data matched the hypothesized models, we applied the diagonally weighted least squares (DWLS) estimation. DWLS is the most suggested CFA method for non-normally distributed data, based on the polychoric correlation matrix (Savalei and Rhemtulla, 2013). We used conventional model fit indices to assess the goodness-of-fit of the acquired CFA models. For the comparative fit index (CFI), we used the recommended cut-off value of >0.90 (Hu and Bentler, 1999). For the root mean square error of approximation (RMSEA), a cut-off value of about 0.08 or less was applied, since this value indicates a reasonable error of approximation. The recommended cut-off value, which should not exceed 0.08, was also used for the standardized root mean square residual (SRMR) (Hu and Bentler, 1999). Exploratory factor analysis (EFA) was used to estimate factor loadings for the first-order factor analysis and second-order factor solution before conducting CFA. The Kaiser-Meyer-Olkin test and Bartlett’s test of sphericity were used as measures of sampling adequacy for factor analysis. We used the following cut-off values: 0.60–0.69, mediocre; 0.70–0.79, average; 0.80–0.89, meritorious; and >0.90, marvellous. For Bartlett’s test of sphericity, the cut-off value was *p* < 0.05 (Kaiser, 1974). Cronbach’s *α* coefficient was calculated to estimate the internal consistency of the CTL-I scales. Values from 0.70 to 0.79 are considered acceptable, those between 0.80 and 0.89 show good reliability, and coefficients approaching 0.90 and above reflect excellent internal consistency (George and Mallery, 2003).

## 3 Results

The normality of the data distribution was proved for every CTL-I scale (with 38 and 41 total items). The *p*-values from the Kolmogorov–Smirnov test with Lilliefors correction (*p* < 0.001) indicate a non-normal distribution for all CTL-I scales (see Table 2). The original 41-item Latvian version of the CTL-I achieved lower reliability levels: *α* = 0.62 for INT (7 items) and *α* = 0.76 for BTR (9 items) and did not reach the level of internal consistency achieved in the original study (see Table 2). Decisions regarding subsequent modifications to the INT (removal of Item 7) and BTR scales (removal of Items 13 and 15) were based on a thorough assessment of three criteria: results from CFA and/or corrected item–total correlation analyses (see the following paragraph), as well as the content and wording of the items. Following these modifications, the reliability of both the INT and BTR scales improved, reaching levels comparable to those reported in the original study.

Internal consistency (Cronbach’s *α*) of the 38 item CTL-I Latvian version scales ranged from 0.73 to 0.92, reflecting good to excellent reliability. This range is consistent with the original study and supports the instrument’s psychometric validity (see Table 2). While INT scored 0.73, indicating an acceptable level of internal consistency, LOM’s value was excellent (α = 0.92), and the remaining four scales achieved a good level (α = 0.81 for BTR, α = 0.82 for GRA, α = 0.81 for CEI, and α = 0.83 for PSP). The internal consistency of the 38-item CTL-I total score in the Latvian sample (α = 0.90) is of a similar magnitude to that reported in the original CTL-I research sample (α = 0.89; Kapusta et al., 2018).

To examine the first-order factor structure of CTL-I, the second research question of the current study, CFA for the Latvian-language six-factor model was conducted. DWLS was used as the recommended CFA method for non-normally distributed data; however, the CFA model retrieved for the 41-item survey did not achieve adequate fit indices. The analysis of modification indices revealed high cross-loadings of items 7, 13, and 15 with several CTL-I factors, as well as multiple residual covariances between these items and other CTL-I items. It was discovered, that INT scale item 7 had a low corrected item total correlation (CITC) coefficient with some items of the same scale (r = 0.02), while items 13 and 15 had statistically appropriate CITC coefficients with most items of the GRA scale (r = 0.32, and r = 0.37). Given that cross-loadings notably affect the performance of the six-factor CFI (Li et al., 2020), following a thorough analysis of both psychometric indices (factor loadings and CITC coefficient) and the items’ content, items 7, 13, and 15 were excluded from the Latvian-language version of the CTL-I. A six-factor solution retrieved from the new 38-item CTL-I demonstrated adequate goodness-of-fit (see Table 3), and the factor loadings, mean values, and CITC coefficients were also within acceptable ranges (see Table 4). It should be noted that in the CTL-I models of other cultures, these items also had relatively lower factor loadings than most other items in the corresponding scales. One reason is that these items have been recoded, but another reason is that their content encompasses different features of these subconstructs than the rest of the corresponding scale item set.

The mutual relations among the INT, BTR, GRA, and CEI scales of 38 item CTL-I Latvian version, tested using Spearman correlation coefficients in the Latvian sample and in the samples of the original study (see Table 5), are statistically significant compared with those between these four factors and PSP and LOM. In the Latvian sample, correlation coefficients between INT, BTR, GRA, and CEI range from 0.36 to 0.61; in the Austrian sample, from 0.70 to 0.91; and in the Polish sample, from 0.69 to 0.90. In the Latvian sample, PSP has a negative statistically significant correlation with INT (r = −0.11, *p* < 0.01), no statistically significant relations with BTR or GRA, and a statistically significant negative relation with CEI (r = −0.10, *p* < 0.01). This differs from previous results, where PSP correlates positively with all CTL-I scales, ranging from 0.15 to 0.49 in the Austrian sample, from 0.17 to 0.39 in the Polish sample, from 0.15 to 0.34 in the Italian sample, and from 0.23 to 0.31 in the Slovenian sample (Kapusta et al., 2018; Margherita et al., 2018; Poštuvan et al., 2022).

In the Latvian, Austrian, Polish, and Slovenian samples, INT and GRA reveal no statistically significant relations with LOM. In contrast, BTR showed no relation with LOM in the Slovenian and Polish samples but acquired a statistically significant, albeit low, correlation (r = 0.21) in the Latvian sample; a similar level of correlation was also observed in the Austrian (r = 0.25) and Italian (r = 0.27) samples. LOM relations with SPS are statistically significant at a level of 0.58 in the Latvian sample, yielding the highest correlation coefficient among all 38 item CTL-I Latvian language scales. Conversely, this relation is statistically significant only at the levels of 0.15 and 0.17 in the Austrian and Polish samples and at the level of 0.24 in the Italian sample; no relations at all were discovered in the Slovenian study (Kapusta et al., 2018; Margherita et al., 2018; Poštuvan et al., 2022).

A comparison of correlation coefficients across all samples (including the Latvian sample) reveals varying strengths of psychometrically significant positive correlations among the CTL-I scales. There is a convincing tendency for the INT, BTR, GRA, and CEI scales to have strong and unambiguous relations, whereas the correlations of these four scales with PSP and LOM scales are significant but weaker, not statistically significant, or even statistically significant and negative relations in the Latvian research sample (see Table 5). In the Latvian sample, the INT and GRA scales did not show statistically significant relations with LOM; although surprising, these results are similar to those from the original study. Moreover, a much stronger correlation was found between PSP and LOM (r = 0.58) than in the original Austrian and Polish samples (r = 0.15 and r = 0.17).

The results of the Spearman correlation analysis strengthened our expectations and encouraged us to examine the probability of a second-order factor structure for the 38 item CTL-I Latvian version. First, EFA second-order analysis for CTL-I scales (based on Eigenvalues greater than 1) was applied, and a very sound two-factor solution for the Latvian CTL-I was obtained, where INT, BTR, GRA, and CEI formed one factor and SPS and LOM formed the other (see Table 6). This second-order EFA model explained 71.16% of the total variance and reached the following goodness-of-fit criteria: CFI = 0.99, RMSEA = 0.05 (90% confidence interval), and SRMR = 0.01 values (see also Table 3).

**Table 3:** Confirmatory factor analysis of CTL-I Latvian version six-factor model and two types of two-factor models.

Model	Factors	*ꭓ* ^2^	*df*	*CFI*	*RMSEA*	*SRMR*
CTL-I	6	1691.179	703	0.95	0.05	0.06
CTL-I^a^	2	54.820	9	0.92	0.08	0.06
CTL-I^b^	2	2696.018	665	0.92	0.08	0.06

^a^Second-order CFA of CTL-I scales.

^b^Second-order CFA of CTL-I items.

**Table 4:** Factor loadings of CTL-I items for first-order six- and two-factor models, item descriptive statistics, and corrected item total correlation coefficients.

CTL-I item	Six-factor model loadings						Item M (SD)	CITC^a^	Two-factor model loadings	
	INT	BTR	GRA	CEI	PSP	LOM			EII^b^	RSDL^c^
1	0.42						3.36 (0.60)	0.36	0.31	
2	0.70						3.21 (0.68)	0.45	0.53	
3	0.60						3.54 (0.60)	0.60	0.42	
4	0.46						3.54 (0.57)	0.51	0.31	
5	0.53						3.35 (0.69)	0.47	0.41	
6	0.53						3.36 (0.64)	0.41	0.43	
8		0.66					3.23 (0.71)	0.58	0.59	
9		0.64					3.03 (0.78)	0.57	0.54	
10		0.72					3.21 (0.77)	0.68	0.61	
11		0.69					3.27 (0.73)	0.67	0.60	
12		0.54					3.42 (0.72)	0.51	0.50	
14		0.49					3.06 (0.80)	0.41	0.40	
16		0.61					3.34 (0.66)	0.46	0.59	
17			0.62				3.47 (0.64)	0.54	0.55	
18			0.60				3.49 (0.62)	0.57	0.54	
19			0.63				3.36 (0.70)	0.58	0.57	
20			0.58				3.40 (0.70)	0.55	0.52	
21			0.69				3.44 (0.67)	0.63	0.61	
22			0.63				3.53 (0.60)	0.59	0.56	
23			0.61				3.39 (0.62)	0.45	0.56	
24				0.74			3.29 (0.70)	0.57	0.69	
25				0.57			3.17 (0.73)	0.51	0.53	
26				0.56			3.38 (0.60)	0.52	0.55	
27				0.52			3.36 (0.63)	0.52	0.49	
28				0.59			3.34 (0.66)	0.61	0.55	
29				0.44			3.44 (0.61)	0.45	0.43	
30				0.59			3.12 (0.89)	0.49	0.54	
31				0.65			3.34 (0.71)	0.55	0.62	
32					0.94		2.71 (0.98)	0.73	0.63	
33					0.77		2.57 (0.78)	0.73	0.52	
34						0.50	2.61 (1.06)	0.45		0.49
35						0.81	2.54 (1.16)	0.76		0.82
36						0.83	2.50 (1.24)	0.77		0.82
37						0.83	2.57 (1.16)	0.81		0.83
38						0.74	2.58 (1.00)	0.72		0.73
39						0.84	2.57 (1.14)	0.80		0.84
40						0.84	2.54 (1.16)	0.81		0.83
41						0.76	2.57 (1.13)	0.44		0.76

^a^Corrected item total correlations.

^b^Emotional investment and intimacy.

^c^Regulation of sexual desire and loss.

**Table 5:** Correlation coefficients of CTL-I scales in Latvian, Austrian, and Polish samples^a^.

Scale	BTR	GRA	CEI	PSP	LOM	CTL-I total
INT	0.36**/0.70***/0.69***	0.40**/0.84***/0.94***	0.41**/0.80***/0.94***	−0.11**/0.40***/0.25**	−0.04/0.11/−0.03	0.41**/0.54***/0.76***
BTR	–	0.48**/0.75***/0.69***	0.56**/0.75***/0.77***	0.07/0.34**/0.22**	0.21**/0.25**/−0.07	0.69**/0.63***/0.69***
GRA		–	0.61**/0.91***/0.90***	−0.04/0.39***/0.30***	0.03/0.04/−0.16	0.56**/0.75***/0.74***
CEI			-	0.10**/0.49***/0.32***	0.18**/0.06/−0.12	0.70**/0.79***/0.80***
PSP				-	0.58**/0.15**/0.17*	0.44**/0.69**/0.66**
LOM					-	0.70**/0.40***/0.28**

Correlations from original CTL-I study (Kapusta et al., 2018).

**Table 6:** Second-order EFA and CFA factor loadings of CTL-I.

CTL-I two-factor solution	Factor loadings				KMO value^a^	Bartlett’s test, p-value
	EFA		CFA			
Emotional investment and intimacy (EII)					0.72	>0.001
Interest in other (INT)	0.61		0.60			
Basic trust (BTR)	0.73		0.73			
Gratitude (GRA)	0.77		0.77			
Common ego ideal (CEI)	0.83		0.84			
Regulation of sexual desire and loss (RSDL)						
Permanence of sexual passion (PSP)		0.74		0.90		
Loss and mourning (LOM)		0.77		0.63		

^a^Kaiser–Meyer–Olkin test value for second-order CTL-I model.

Thus, we proceeded with a CFA of the second-order model. The acquired Kaiser-Meyer-Olkin value of 0.72 and the Bartlett’s test results for second-order EFA analysis (ꭓ2 = 1282.951, df = 15.000, *p* < 0.001) suggested that the factors were suitable for factor analysis. After analyzing the item-level content of the newly obtained two-factor solutions, the two factors were named emotional investment and intimacy (EII) and regulation of sexual desire and loss (RSDL). We ran one more CFA for the new two-factor solution (on an item level), entering all 38 items of the Latvian CTL-I: 28 items from INT, BTR, GRA, and CEI for the first newly obtained factor (EII) and 10 items from PSP and LOM for the second factor (RSDL) (see Table 3 for model fit indices and Table 7 for factor loadings).

**Table 7:** Factor loadings of CTL-I items of two-factor solution, item descriptive statistics, and corrected item total correlation coefficients.

CTL-I Item (Original)	CTL-I (LV) item	Factor loadings		Item M (SD)	Corrected item total correlation
		EII	SSR		
Emotional investment and intimacy (EII)					
1	1	0.31		3.36 (0.59)	0.32
2	2	0.53		3.22 (0.67)	0.52
3	3	0.42		3.54 (0.60)	0.42
4	4	0.31		3.54 (0.56)	0.30
5	5	0.41		3.35 (0.68)	0.40
6	6	0.43		3.35 (0.64)	0.44
8	7	0.59		3.24 (0.70)	0.56
9	8	0.54		3.03 (0.78)	0.51
10	9	0.61		3.20 (0.78)	0.59
11	10	0.60		3.27 (0.73)	0.57
12	11	0.50		3.41 (0.72)	0.48
14	12	0.40		3.05 (0.80)	0.38
16	13	0.59		3.34 (0.66)	0.56
17	14	0.55		3.47 (0.64)	0.53
18	15	0.54		3.48 (0.62)	0.52
19	16	0.57		3.36 (0.71)	0.54
20	17	0.52		3.39 (0.70)	0.48
21	18	0.61		3.43 (0.67)	0.57
22	19	0.56		3.53 (0.60)	0.53
23	20	0.56		3.40 (0.62)	0.55
24	21	0.69		3.29 (0.70)	0.64
25	22	0.53		3.16 (0.73)	0.49
26	23	0.55		3.38 (0.60)	0.52
27	24	0.49		3.36 (0.63)	0.46
28	25	0.55		3.34 (0.66)	0.53
29	26	0.43		3.44 (0.61)	0.42
30	27	0.54		3.11 (0.89)	0.50
31	28	0.62		3.33 (0.72)	0.57
Regulation of sexual desire and loss (RSDL)					
32	29		0.63	2.70 (0.98)	0.64
33	30		0.52	2.56 (0.77)	0.53
34	31		0.49	2.60 (1.06)	0.48
35	32		0.82	2.52 (1.15)	0.79
36	33		0.82	2.47 (1.23)	0.79
37	34		0.83	2.55 (1.16)	0.79
38	35		0.73	2.57 (1.01)	0.71
39	36		0.84	2.55 (1.14)	0.79
40	37		0.83	2.53 (1.15)	0.79
41	38		0.76	2.56 (1.15)	0.72

The inter-scale relations of the Latvian CTL-I’s primary and secondary scales reveal that INT, BTR, GRA, and CEI correlate with EII in a range from 0.62 to 0.84. However, PSP does not show significant relations with EII, and LOM has a statistically significant relation with EII at the level of 0.16 (*p* < 0.01). Simultaneously, SPS and LOM have a statistically significant relation with RSDL (r = 0.69 and r = 0.98). EII and RSDL’s mutual relations are statistically significant, although slightly lower at 0.15 (p < 0.01) (see Table 8 for all correlation coefficients).

**Table 8:** Correlation coefficients of CTL-I primary and second-order scales.

Scales	EII	RSDL	CTL-I total
Primary scales			
Interest in the other (INT)	0.62**	−0.06	0.41**
Basic trust (BTR)	0.80**	0.20**	0.69**
Gratitude (GRA)	0.78**	0.02	0.56**
Common ego ideal (CEI)	0.84**	0.17**	0.70**
Permanence of sexual passion (PSP)	−0.03	0.70**	0.44**
Loss and mourning (LOM)	0.16**	0.98**	0.70**
Second-order scales			
Emotional investment and intimacy (EII)		0.15**	0.78**
Regulation of sexual desire and loss (RSDL)			0.70**

## 4 Discussion

The current study examined the internal consistency and several psychometrically valid factor structures of the CTL-I following its adaptation into Latvian in a non-clinical sample. The first research question was whether the reliability of the CTL-I scales in the Latvian-language version was similar to that in the original study. Reliability coefficients indicated that the internal consistency of all subscales and the total score of the 38-item Latvian version of the CTL-I was adequate to excellent. The reliability values were comparable to those reported for the original CTL-I. However, the INT and BTR scales required revision, as three items (Items 7, 13, and 15) showed psychometric divergence and content ambiguity relative to the other items within their respective scales.

*The Guidelines for Translating and Adapting Tests* (International Test Commission, 2017) suggest that the application of instruments in a new linguistic or cultural context may reveal issues that have not previously been found. Accordingly, when we analyzed the content of item 7 (“I am often bored with my partner”), which is scored reversely, contextually, it does not fully align with the common reality of the INT scale. While the content of the scale’s other six items characterizes people’s affection, empathy, and willingness to share emotional experiences, the statement about boredom brings in a different subject. Kernberg (2011) states that boredom with the subjective experience of the other, as well as with the experience of the relationship as more ‘transactional’ than interpersonal, ultimately signals a tendency to take a partner for granted, which is typical of personalities with narcissistic character traits. Although boredom is a very important topic (especially in clinical populations), in the non-clinical Latvian population, it was clearly inconsistent with the concept conveyed by the other INT scale items. The authors of the present study suggest that the issue with Item 7, which diverges from the INT scale, may reflect an age-related phenomenon among young adults (≤ 35 years), who comprised the majority of the Latvian sample. During this period, individuals are also engaged in other developmental tasks, such as consolidating an adult identity and progressing along the “ladder” of becoming fully integrated adult members of society–also in workplace and community (Shulman and Connolly, 2013), additonally the authors assume, that less stable social and economic conditions may contribute to the delayed establishment of deeply committed romantic relationships.

Similarly, items 13 and 15 were excluded from the Latvian version of the BTR scale because the factor analysis revealed high cross-loadings with several CTL-I factors and multiple residual covariances with other items. The low factor loadings of items 13 and 15 in the Latvian sample, as well as in several previous studies (Kapusta et al., 2018; Margherita et al., 2018; Poštuvan et al., 2022), prompted the authors of the current research to analyze their content to estimate whether these items dissonate with other items on the BTR scale. The statements “I keep secrets from my partner” (item 13) and “I sometimes feel that my relationship is limited” (item 15), which again are both scored reversely, bring two additional aspects to the BTR scale–the topic of secrets and the acknowledgement of limitations. This diverges from the other BTR items, which focus on one’s ability to trust and on one’s willingness and capacity to enjoy the emotional intimacy and mutual revelations of the intimate aspects of one’s inner life (Kapusta et al., 2018).

The INT scale also revealed lower and more diverse factor loadings for the scale items obtained from CFA in the Austrian and Polish studies (Kapusta et al., 2018), as well as from the Slovenian sample (Poštuvan et al., 2022), in comparison to the other CTL-I scales. Item 7 also showed lower factor loadings in the Italian version of the CTL-I (Margherita et al., 2018). Therefore, the authors of this study were interested in how items 7, 13, and 15 function in other cultural environments, since in the Latvian sample, they signal a certain dissonance. According to *The Guidelines for Translating and Adapting Tests* (International Test Commission, 2017), if cultural groups differ on important variables that are irrelevant to the construct being measured, comprehensive designs and statistical analyses can be used to control for these “nuisance” variables. Moreover, the “inductive–lexical–trait perspective,” which is the fundamental principle for the study of personality traits, holds that the most important dimensions of personality are encoded in language (Davis et al., 2018). Therefore, the authors of the current research assume that these “problematic” items may reveal clinically relevant aspects. Items 7, 13, and 15 include contextual references to the negative end of the CTL-I construct, that is, limitations to the capacity to love–namely, feelings of boredom, a tendency to keep secrets, and feelings of limitedness in the romantic partnership. Quantitative research on these items should be continued, since a person who admits to these problematic feelings and behavioral dispositions may be able to regulate these undesirable emotional states, rather than being overwhelmed by them. The age range of the present sample may have contributed to variability in responses to Items 13 and 15, as younger individuals typically have less experience in romantic relationships and are still engaged in other key developmental tasks (Arnett, 2000; Shulman and Connolly, 2013). Cultural factors may also influence responses to Item 13, with potential differences across Italian, Portuguese, Polish, Slovenian, and Latvian participants. For example, Latvian cultural norms are often characterized as more introverted, which may shape item interpretation; however, this remains speculative and requires further empirical investigation. In addition, the predominance of female participants may have contributed to the divergence of Item 15 from the BTR scale, as women may be more attuned to relationship difficulties and more likely to anticipate potential break-up threats earlier than men (Blumstein and Schwartz, 1983).

The second research question was whether the original six-factor structure of the CTL-I would be confirmed in the Latvian population with acceptable values of goodness-of-fit indices. The original CTL-I inventory (41 items) was not replicated in the Latvian population with acceptable goodness-of-fit values in its original six-factor structure. The analysis of the acquired model revealed high item residual covariances and cross-loadings with other factors, and, accordingly, low factor loadings. Items 7, 13, and 15 had a tendency to migrate across several CTL-I scales. When these items were excluded from the factor matrix, the six-factor structure of the 38-item CTL-I Latvian version achieved the necessary levels of goodness-of-fit indices. Thus, the second research question reached a psychometrically supported resolution, and a six-factor structure for the Latvian version of the CTL-I was achieved. Accordingly, the results of this study support the use of the 38-item Latvian version of the CTL-I (Table 7).

The results of the inter-scale correlation analysis, supported by the current study, served as the basis for the third research question: whether the six-factor CTL-I model also reveals a two-factor second-order solution in the Latvian sample, with the theoretical construct of the contextual logic of capacity to love and adequate psychometric criteria for model fit. As predicted, the results of the second-order factor analysis revealed that the INT, BTR, GRA, and CEI scales form one factor (EII), while PSP and LOM form a second factor (RSDL). The names for these newly discovered factors were chosen according to the theoretical construct of capacity for love (Kernberg, 2011), which forms part of Kernberg’s wider model of developmental psychopathology Kernberg (1974, 1988). Kernberg (2011) states that the search for reconfirmation of intimacy is a healthy counterpart to opportunistic criteria regarding the advantages of staying with a romantic partner. Conversely, Kernberg (2007) describes the capacity for affective investment as a basic precondition for establishing a healthy object relationship as a counterpart to any inability to invest in an emotional bond with another person, which is typical of narcissistic and other character pathologies. RSDL unites the SPS and LOM subscales. A comprehensive analysis of the SPS and LOM scales’ statements revealed that the SPS scale addresses one’s regulatory ability to deal with difficult emotional and physiological phenomena. The statements “Sexual boredom arises in long-term sexual relationships” and “Sexual desire diminishes throughout time” (Kapusta et al., 2018) touch on the complicated realm of romantic partnerships. Kernberg (2011) states that the development of sexual boredom in a long-term, lasting relationship is a widespread symptom of character pathologies. A persisting syndrome of the desexualization of romantic partners and new manic idealization of sexually exciting ones is the most frequent expression of a combination of unresolved Oedipal conflicts and narcissistic pathology (Kernberg, 2011). This was also proved quantitatively by Kapusta et al. (2018), who demonstrated the inverted relations between CTL and sociosexuality (readiness to engage in an uncommitted sexual relationship). Since divergent reliability was not the aim of the current research, these questions should be examined in future research with Latvian samples.

Across five samples (original Austrian/Polish, Italian, Slovenian, and Latvian), the pattern of correlations indicates that the core CTL-I dimensions (INT, BTR, GRA, CEI) are moderately to strongly interrelated, whereas their associations with PSP and particularly LOM are weaker and less consistent, supporting a partial structural differentiation within the construct. In the current research, the INT and GRA scales have statistically significant negative relations with PSP, whereas BTR and CEI reveal no relations with SPS. Evidence from all previous study samples shows small but generally positive associations between PSP with the core dimensions in the Austrian/Polish, Italian, and Slovenian samples (Kapusta et al., 2018; Margherita et al., 2018; Poštuvan et al., 2022).

In contrast, LOM demonstrates the greatest variability, with predominantly weak or non-significant relations to the core scales across studies (Kapusta et al., 2018; Poštuvan et al., 2022); however, the Latvian findings reveal a comparatively strong association with PSP, suggesting specific linkage between passion and loss-related processes. The unprecedented correlation pattern between LOM and PSP, further supported by the second-order factor structure, may be partly explained by the characteristics of the sample - namely, the predominance of young adults (≤ 35 years). This group corresponds to the novice phase (Levinson et al., 1978) or the transitional emerging adulthood romantic stage (Shulman and Connolly, 2013). Thus, it is plausible that romantic relationships in this developmental period are experienced with greater emotional intensity and identity investment, leading to a closer coupling between two scales representing affectively stronger domains—sexuality and separation-related distress (Arnett, 2000; Shulman and Connolly, 2013). The strong overrepresentation of women may further contribute to this pattern, as women tend to report higher relational interdependence and sensitivity to loss, which can strengthen associations between passionate involvement and mourning processes (Cross and Madson, 1997).

A significant discovery is the lack of significant relations between SPS and the newly discovered EII, which could be discussed in light of Kernberg’s (2011) clinical observation that sexual indifference and boredom are the most immediate manifestations of a lack of contact with deeper emotional and sexual needs. The authors predict that the inter-scale and inter-factor relations revealed by this study could yield new insights when research on CTL-I in clinical groups takes place. As Kernberg (2001) stresses, the unconscious fantasy is more important than fluctuations in the hormonal level or biological status in determining the intensity of sexual excitement and erotic desire. The LOM subscale addresses other intense affects, such as jealousy, a desire for revenge, or feeling unworthy after separation from a partner or during an unreciprocated romantic situation (Kapusta et al., 2018). Thus, merging the SPS and LOM scales into a single factor, as suggested by the second-order factor analysis in the Latvian sample, seems very logical, since both scales deal with affectively loaded and emotionally and physiologically difficult-to-bear states. Therefore, the authors employed the term “regulation” in the name for the second newly discovered second-order factor (RSDL), which is used in affect regulation and management of inner conflict. These terms are repeatedly used in the clinical theory of Kernberg and his team (Diamond et al., 2014).

The emergence of a second-order factor structure, distinguishing emotional investment and intimacy (EII) from regulation of sexual desire and loss (RSDL), may be interpreted in light of other prominent love constructs, for example Sternberg’s Triangular Theory of Love (Sternberg, 1986). The EII factor appears to align with Sternberg’s intimacy and commitment components, reflecting relatively stable emotional closeness and mutual investment. In contrast, RSDL may capture aspects related to passion, albeit in a broader and more regulatory sense. However, these interpretations remain tentative and should be examined in future research, for example, through the assessment of convergent validity.

Social and economic pressures experienced by emerging adults in Western industrialized societies are associated with delayed commitment to romantic partnerships, marriage, and parenthood (Arnett, 2000; Shulman and Connolly, 2013). In the European Union, the mean age at first marriage has risen to the mid-30s for men and the early-to-mid-30s for women (Eurostat, 2024). These shifts may reflect broader changes in romantic love, including its cognitive, behavioral, emotional, and sexual dimensions.

The researchers predict that this RSDL and EII factor structure could potentially provide stronger discriminant power for measuring CTL, but this requires further examination. While EII combines the scales and items that shed light on one’s capacity for emotional intimacy and emotional investments, RSDL combines statements about one’s capacity to meet the emotional and sexual challenges of romantic relationships. Presumably, this two-factor structure allows for a better understanding of the CTL-I instrument’s assessment purpose across non-clinical and clinical populations. Moreover, this study found a low (albeit significant) correlation between the two new sub-constructs in the Latvian non-clinical sample. It is possible to speculate that if the relations between EII and RSDL vary among individuals in the general population, then even greater diversity could be expected in clinical groups. Since this expanded second-level two-factor model was discovered during this CTL-I adaptation study, it should be evaluated in greater depth in future research among the Latvian population. There is also a possibility that it is not relevant in other cultural environments.

### 4.1 Limitations

A limitation of this study is the omission of items 7, 13, and 15 from the adapted Latvian version of the CTL-I. As a result, the shortened instrument may be less comparable to the original and less sensitive to subtle differences. However, within this study, the removal of ambiguous items improved internal consistency and the soundness of the factor structure.

The imbalance in the study sample - specifically, the predominance of women (approximately 80%) and the overrepresentation of participants aged 35 years or younger (85%) - may limit the generalizability of the findings. In supplementary analyses the authors of current reseach examined first - and second-order factor solutions of the 38-item Latvian version of the CTL-I in two subsamples (≤ 35 years, n = 552; 35–60 years, n = 97): although acceptable goodness-of-fit indices were observed, these results are not reported due to the relatively small size of the older subsample.

Future research would benefit from more balanced gender and age distributions to evaluate the variability of CTL scales and the stability of the factor structure across groups stratified by gender, age, partnership status, and romantic relationship experience. The absence of established convergent and discriminant validity for the Latvian CTL-I may further constrain the conclusions that can be drawn regarding the CTL construct. However, divergent validity and associations with maladaptive personality traits in a Latvian sample have been examined by the researchers of actual study and will be reported separately. Nevertheles, additional research is needed to establish convergent validity with other romantic love measures, particularly in relation to the newly identified second-order factor structure. Further work is also required to examine associations with adaptive and maladaptive personality traits and to establish clinical validity across diverse diagnostic groups, as well as to evaluate the extended structural model in other sociocultural samples.

### 4.2 Implications for practice

The current study demonstrated the factorial validity and internal consistency of the CTL-I instrument in a sample from a different cultural environment. The 38-item CTL-I Latvian version of the provides additional evidence that it is a scientifically grounded tool for measuring a person’s capacity to be involved in and to sustain a romantic partnership. The CTL-I’s original, six-factor structure was also supported for the Latvian version. The novelty of the current study lies in its discovery of a second-order factor structure for the CTL-I, with two newly identified factors–EII and RSDL. This two-factor structure potentially provide deepened understanding of the concept of CTL, gained by the use of factor analysis, which is currently the leading scientific methodology for the examination of personality traits (Davis et al., 2018). A deeper understanding of the CTL-I’s latent structure would enhance its accuracy of use, since different people may have different relations between these two factors. Although the findings of current study require replication and additional evidence, authors suggest that the new second-order factor structure may enhance the detection of personality problems related to romantic love issues. The CTL-I may be employed as an adjunctive assessment tool, particularly in cases of problematic romantic relationships, given that CTL has potential utility as a prognostic construct in psychotherapeutic treatment (Kernberg, 1974).

The International Test Commission (2001) advises that when psychological tests are applied, researchers and clinicians should have a good understanding of the scales used, the characteristics of the norm or comparison groups, and the limitations of the scores, therefore future research should further analyze the problematic CTL-I items cross-culturally, establish convergent, divergent and clinical validity, and follow guidelines for test use–clarifying norms, scale properties, and score limitations.

## Data Availability

The raw data supporting the conclusions of this article will be made available by the authors, without undue reservation.

## References

[ref1] Arnett J. J. (2000). Emerging adulthood: A theory of development from the late teens through the twenties. Am. Psychol. 55, 469–480. doi: 10.1037/0003-066X.55.5.469, 10842426

[ref2] Blumstein P. Schwartz P. (1983). American Couples: Money, Work, Sex. New York: William Morrow and Company.

[ref3] Cross S. E. Madson L. (1997). Models of the self: self-construals and gender. Psychol. Bull. 122, 5–37. 9204777 10.1037/0033-2909.122.1.5

[ref4] Davis R. D. Samaco Zamora M. C. Millon T. (2018). “Theoretical versus inductive approaches to contemporary personality pathology,” in Handbook of Personality Disorders: Theory, Research, and Treatment, ed. Livesley W. J.. 2nd ed (New York: Guilford Press), 25–45.

[ref5] Diamond D. Clarkin J. F. Levy K. N. Meehan K. B. Cain N. M. Yeomans F. E. . (2014). Change in attachment and reflective function in borderline patients with and without comorbid narcissistic personality disorder in transference focused psychotherapy. Contemp. Psychoanal. 50, 175–210. doi: 10.1080/00107530.2014.880316

[ref6] Eurostat (2024) in Mean age at first Marriage by sex (Dataset code: tps00014), ed. European Commission.

[ref7] Fonte C. Campos C. Ferreira M. J. (2022). Estudo preliminar de adaptação e validação para Portugal do Inventário de Capacidade para amar [Poster presentation]. 5° Congresso da Ordem dos Psicólogos Portugueses. Aveiro, Portugal: Ordem dos Psicólogos Portugueses.

[ref8] Fonte C. Pires S. Ferreira M. J. (2025). The role of personality in the capacity to love. Int. J. Psychol. Res. 18, 69–81. doi: 10.21500/20112084.6843, 41001356 PMC12410218

[ref9] Freud S. (1957). “Mourning and melancholia,” in The Standard edition of the Complete Psychological Works of Sigmund Freud, ed. Strachey J., vol. 14 (London: Hogarth Press), 237–258.

[ref10] Freud S. (1959). “Delusions and dreams in Jensen’s Gradiva,” in The Standard edition of the Complete Psychological Works of Sigmund Freud, ed. Strachey J. (London: Hogarth Press).

[ref11] Fuchshuber J. Neugebauer J. Blüml V. Kapusta N. D. (2025). The relationship between attachment, the capacity to love, and relationship satisfaction: a mediation analysis. Psychoanal. Psychol. 42, 159–163. doi: 10.1037/pap0000537

[ref7001] George D. Mallery P. (2003). SPSS for Windows step by step: A simple guide and reference. (4th ed.). Boston: Allyn & Bacon.

[ref12] Hatfield E. Sprecher S. (1986). Measuring passionate love in intimate relationships. J. Adolesc. 9, 383–410. doi: 10.1016/S0140-1971(86)80043-4, 3805440

[ref13] Hendrick S. S. (1988). A generic measure of relationship satisfaction. J. Marriage Fam. 50, 93–98. doi: 10.2307/352430

[ref14] Hu L. T. Bentler P. M. (1999). Cutoff criteria for fit indexes in covariance structure analysis: conventional criteria versus new alternatives. Struct. Equ. Model. 6, 1–55. doi: 10.1080/10705519909540118

[ref15] International Test Commission (2001). International guidelines for test use. Int. J. Test. 1, 93–114. doi: 10.1207/S15327574IJT0102_1

[ref16] International Test Commission (2017). The ITC Guidelines for Translating and Adapting Tests. 2nd Edn https://www.intestcom.org.

[ref17] Kaiser H. F. (1974). An index of factorial simplicity. Psychometrika 39, 31–36. doi: 10.1007/BF02291575

[ref18] Kapusta N. D. Jankowski K. S. Wolf V. Chéron-Le Guludec M. Lopatka M. Hammerer C. . (2018). Measuring the capacity to love: development of the CTL-inventory. Front. Psychol. 9:1115. doi: 10.3389/fpsyg.2018.01115, 30087627 PMC6066550

[ref19] Kernberg O. F. (1974). Barriers to falling and remaining in love. J. Am. Psychoanal. Assoc. 22, 486–511. doi: 10.1177/000306517402200302, 4455724

[ref20] Kernberg O. F. (1977). Boundaries and structure in love relations. J. Am. Psychoanal. Assoc. 25, 81–114. doi: 10.1177/000306517702500104, 858903

[ref21] Kernberg O. F. (1988). Psychic structure and structural change: an ego psychology–object relations theory viewpoint. J. Am. Psychoanal. Assoc. 36, 315–337.

[ref22] Kernberg O. F. (2001). Object relations, affects, and drives: toward a new synthesis. Psychoanal. Inq. 21, 604–619. doi: 10.1080/07351692109348963

[ref23] Kernberg O. F. (2007). The almost untreatable narcissistic patient. J. Am. Psychoanal. Assoc. 55, 503–539. doi: 10.1177/00030651070550020701, 17601104

[ref24] Kernberg O. F. (2011). Limitations to the capacity to love. Int. J. Psychoanal. 92, 1501–1515. doi: 10.1111/j.1745-8315.2011.00456.x, 22212039

[ref25] Kline R. B. (2023). Principles and Practice of Structural Equation Modeling. New York, NY: Guilford Publications.

[ref7002] Levinson D. J. Darrow C. N. Klein E. B. Levinson M. H. McKee B. (1978). The seasons of a man’s life. New York, NY: Random House.

[ref26] Li Y. Wen Z. Hau K.-T. Yuan K.-H. Peng Y. (2020). Effects of cross-loadings on determining the number of factors to retain. Struct. Equ. Model. 27, 841–863. doi: 10.1080/10705511.2020.1745075

[ref27] Margherita G. Gargiulo A. Troisi G. Tessitore F. Kapusta N. D. (2018). Italian validation of the capacity to love inventory: preliminary results. Front. Psychol. 9:1434. doi: 10.3389/fpsyg.2018.01434, 30158888 PMC6104494

[ref28] Poštuvan V. Glavač T. Schmeckenbecher J. Kapusta N. D. (2022). Slovenian validation of the capacity to love inventory – a preliminary study. Psychol. Top. 31, 317–335. doi: 10.31820/pt.31.2.6 PMC11417625 39315040

[ref29] Quispilaya-Capcha K. L. Yupanqui-Lorenzo D. E. Montes-Herrera G. Romero-Alvarez F. M. Arauco-Lozada T. (2024). The relationship assessment scale revised (Ras-R): adaptation and psychometric evaluation by classical test theory and item response theory. TPM Test. Psychom. Methodol. Appl. Psychol. 31, 271–284. doi: 10.4473/TPM31.3.1

[ref30] Rubin Z. (1970). Measurement of romantic love. J. Pers. Soc. Psychol. 16, 265–273. doi: 10.1037/h0029841, 5479131

[ref31] Savalei V. Rhemtulla M. (2013). The performance of robust test statistics with categorical data. Br. J. Math. Stat. Psychol. 66, 201–223. doi: 10.1111/j.2044-8317.2012.02049.x, 22568535

[ref32] Shulman S. Connolly J. (2013). The challenge of romantic relationships in emerging adulthood. Emerg. Adulthood 1, 27–39. doi: 10.1177/2167696812467330

[ref33] Sternberg R. J. (1986). A triangular theory of love. Psychol. Rev. 93, 119–135. doi: 10.1037/0033-295X.93.2.119

